# Conservative Management of Cervicogenic Dizziness Associated With Upper Cervical Instability and Postural Orthostatic Tachycardia Syndrome: A Case Report

**DOI:** 10.7759/cureus.72765

**Published:** 2024-10-31

**Authors:** Robert J Trager, Andres Schuster, Cliff Tao, Gina Zamary

**Affiliations:** 1 Chiropractic Medicine, Connor Whole Health, University Hospitals Cleveland Medical Center, Cleveland, USA; 2 Family Medicine and Community Health, Case Western Reserve University School of Medicine, Cleveland, USA; 3 Biostatistics and Bioinformatics Clinical Research Training Program, Duke University School of Medicine, Durham, USA; 4 College of Chiropractic, Logan University, Chesterfield, USA; 5 Harrington Heart and Vascular Institute, University Hospitals Cleveland Medical Center, Cleveland, USA; 6 Radiology, Private Practice of Chiropractic Radiology, Irvine, USA; 7 Connor Whole Health, University Hospitals Cleveland Medical Center, Cleveland, USA

**Keywords:** case reports, cervical vertebrae, chiropractic, dizziness, headache, joint instability, neck pain, postural orthostatic tachycardia syndrome, rehabilitation

## Abstract

Cervicogenic dizziness (CGD) is a disorder in which dizziness arises from cervical spine dysfunction and is diagnosed after excluding other conditions. We present a case of a 27-year-old woman with a six-year history of dizziness, neck and cervicothoracic pain, and occipital-temporal-orbital headaches. The patient also experienced occasional severe, incapacitating episodes of dizziness with vomiting. Previous evaluations, including advanced imaging, had helped rule out central, otolithic, and psychogenic causes of dizziness. Dynamic radiographs revealed signs of lateral instability of C1 while tilt table testing revealed postural orthostatic tachycardia syndrome (POTS). Over eight months, the patient underwent a regimen of gentle manual therapies and cervical stability exercises. The patient’s dizziness substantially improved, as measured by the Dizziness Handicap Inventory (DHI), with scores reducing from 50 (moderate handicap) to 10 (less than mild handicap). This case highlights the importance of considering cervical spine dysfunction and associated conditions like POTS in the differential diagnosis of chronic dizziness. While conservative management including manual therapy appeared effective in this case for CGD with underlying upper cervical instability and POTS, additional research is needed on this topic.

## Introduction

Dizziness is a non-specific term used to describe various sensations, including lightheadedness, unsteadiness, or a spinning feeling. Its utility as a diagnostic term is limited due to its broad nature, necessitating a thorough assessment of the patient’s history and examination for an accurate diagnosis [1]. Dizziness is typically evaluated based on its timing, associated triggers, and symptom subtypes. For instance, benign paroxysmal positional vertigo (BPPV), the most common cause of dizziness, accounting for approximately 24% of cases, presents with brief episodes of rotatory dizziness triggered by head movements [1,2]. Other subtypes of dizziness may arise from vestibular, vascular, or psychogenic sources, with less frequent causes including Meniere’s disease, vestibular neuritis, and others [2].

Cervicogenic dizziness (CGD) is defined as a clinical syndrome in which dizziness arises from dysfunction in the cervical spine [3]. Some of the proposed mechanisms of CGD include disc herniations, spondylosis, and upper cervical instability (UCI) at the C1-C2 level [4-6]. However, diagnosing CGD remains challenging due to the absence of consensus diagnostic criteria and validated tests, making clinical judgment and the exclusion of other causes the primary diagnostic approach [3,7]. Unlike other forms of dizziness, CGD typically presents with longer-lasting episodes, with symptoms persisting for minutes to hours [7]. Among adults with neck pain, the prevalence of CGD is estimated to be approximately 8.1% [4].

We present the case of a 27-year-old woman with a six-year history of chronic dizziness, headaches, and neck pain, ultimately diagnosed with CGD associated with C1 lateral instability and underlying postural orthostatic tachycardia syndrome (POTS). This report highlights the importance of recognizing CGD as a differential diagnosis in patients with chronic dizziness and illustrates the clinical improvement following multidisciplinary conservative management.

## Case presentation

A 27-year-old woman presented to a chiropractor and cardiologist in 2024 with over six years of dizziness, neck and cervicothoracic muscle tightness and pain, and occipital, temporal, and orbital headaches. Symptoms began suddenly in early 2018 after sleeping on a mattress with an uncomfortable support rod. In the six years preceding this event, she was in a motor vehicle collision, sustained a concussion while playing soccer that caused temporary dizziness, and had a molar infection and underwent a root canal procedure requiring amoxicillin. She previously played high school soccer and had regularly practiced header drills (i.e., using the head to pass, shoot, or clear the ball). Her General Anxiety Disorder-7 (GAD-7) score was 14 (moderate anxiety) while her Patient Health Questionnaire-9 (PHQ-9) score was 7 (mild depressive symptoms). She described much of her anxiety to a concern about triggering presyncope/syncope or severe dizziness. Her Dizziness Handicap Inventory (DHI) score was 50 (mild handicap, 16-34; moderate handicap, 36-52; severe handicap, 54+), indicating a moderate dizziness handicap. The patient had recently moved to complete a post-doctoral fellowship.

The patient described the dizziness as frequent lightheadedness with occasional near syncope, without distinct spinning. She reported long-lasting episodes once or twice per year where any movement would provoke dizziness to the degree that she would vomit and was bedridden. The neck pain was mild but constant, prompting the patient to massage her suboccipital regions regularly. Dizziness was worsened by hunger, walking down the supermarket aisles, quick head movements, and sometimes bending over. She reported that these symptoms substantially limited her quality of life. She avoided supermarkets and shopping alone, would only drive in the slow lane on highways in case she had to pull over, had difficulty cooking due to dizziness, and had difficulty eating due to nausea. She noted concerns of prolonged standing in lines due to the near syncope.

**Figure 1:**
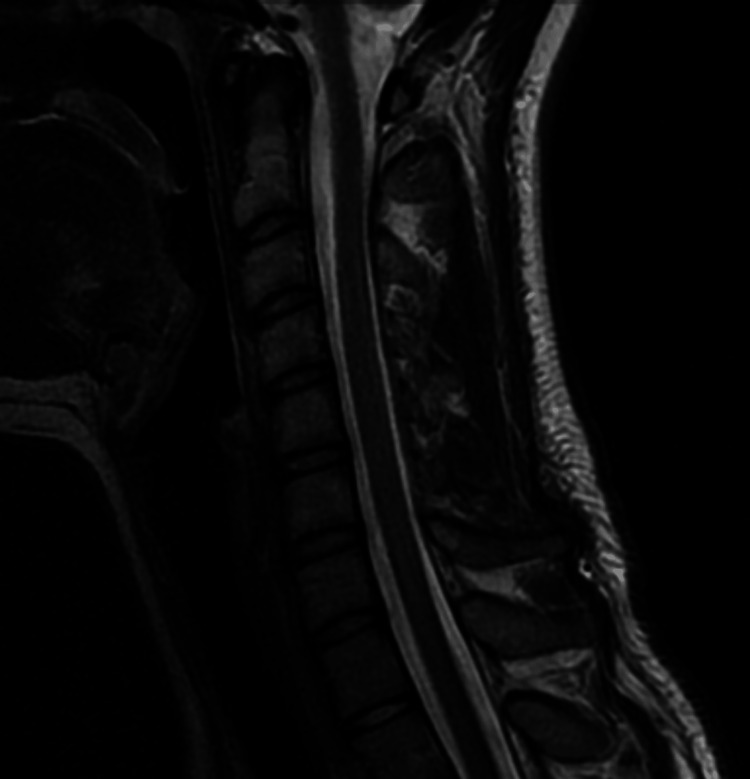
Cervical spine magnetic resonance imaging A sagittal T2-weighted image. There are no evident abnormal findings.

Previously, a neurologist suspected multiple sclerosis (2019); however, brain and cervical magnetic resonance imaging without/with contrast and her clinical features did not support the diagnosis (Figure 1). An otorhinolaryngologist suspected BPPV yet vestibular exercises did not provide relief. Clinicians also suspected anxiety as a trigger yet selective serotonin reuptake inhibitors (fluoxetine, escitalopram) and bupropion did not alleviate symptoms. A prior cardiologist suspected POTS and recommended increased salt intake and hydration. Fludrocortisone (0.1 mg) had only slightly alleviated her symptoms, and the patient had discontinued this medication. A psychiatrist suspected panic disorder and prescribed venlafaxine (150 mg), which did not alleviate the patient’s dizziness. The patient’s family history was relevant for bipolar depression (parent).

Upon presenting to our healthcare organization, she visited a cardiologist due to occasional heart palpitations. Upon examination, her blood pressure was 105/70 mmHg, heart rate was 73 bpm, and body mass index was 24.81 kg/m². There were normal S1 and S2 heart sounds without murmurs, and the extremities were free of peripheral edema with 2+ pulses bilaterally. The electrocardiogram (ECG) revealed a sinus rhythm at 67 bpm with normal atrioventricular conduction and ventricular repolarization. Echocardiography indicated normal left ventricular systolic function, with an estimated ejection fraction of 55-60%. The cardiologist initially suspected dysautonomia and/or vagal involvement due to a previous concussion.

Consequently, the cardiologist obtained autonomic testing. The tilt table test revealed exaggerated orthostatic tachycardia with a rise in heart rate from 67 to 112 beats per minute. Other tests including responses to deep breathing, the Valsalva maneuver, and the sudomotor axon reflex were within normal limits [8]. The cardiologist concluded that the findings were consistent with POTS and recommended increasing salt and water intake. The patient changed her dietary habits to include more salty foods as snacks. Considering the assessment showed no evidence of structural cardiovascular abnormalities, the cardiologist also recommended follow-up with a chiropractor to address potentially related cervical spine dysfunction, given the patient’s past injuries.

An examination by the chiropractor revealed a normal cervical range of motion with pain and hypertonic and tender suboccipital muscles, cervical erectors, upper trapezii, and temporomandibular muscles. There was a soft end feel in the upper cervical region during motion palpation and provocative ligamentous tests were therefore avoided. Cranial nerves 2 through 12 were intact, and the patient’s coordination, motor strength, sensation, and muscle stretch reflexes were within normal limits. Romberg’s test and Fukuda’s stepping test were normal. Pathologic reflexes (including those of Hoffmann (via finger flick) and Rossolimo (via plantar tap)) were absent. During a bedside vestibular oculomotor screening test, tests for the horizontal and vertical vestibular-ocular reflex, in which the patient maintains a fixed gaze on a target while actively moving their head, exacerbated the patient’s dizziness. Additionally, the visual motion sensitivity test, in which the patient fixes their gaze on their outstretched thumb while rotating their torso side to side, also provoked dizziness. No features within the Beighton scale were present (i.e., a nine-point scoring system to assess general hypermobility via physical tests including the thumbs, elbows, knees, and forward bending).

**Figure 2:**
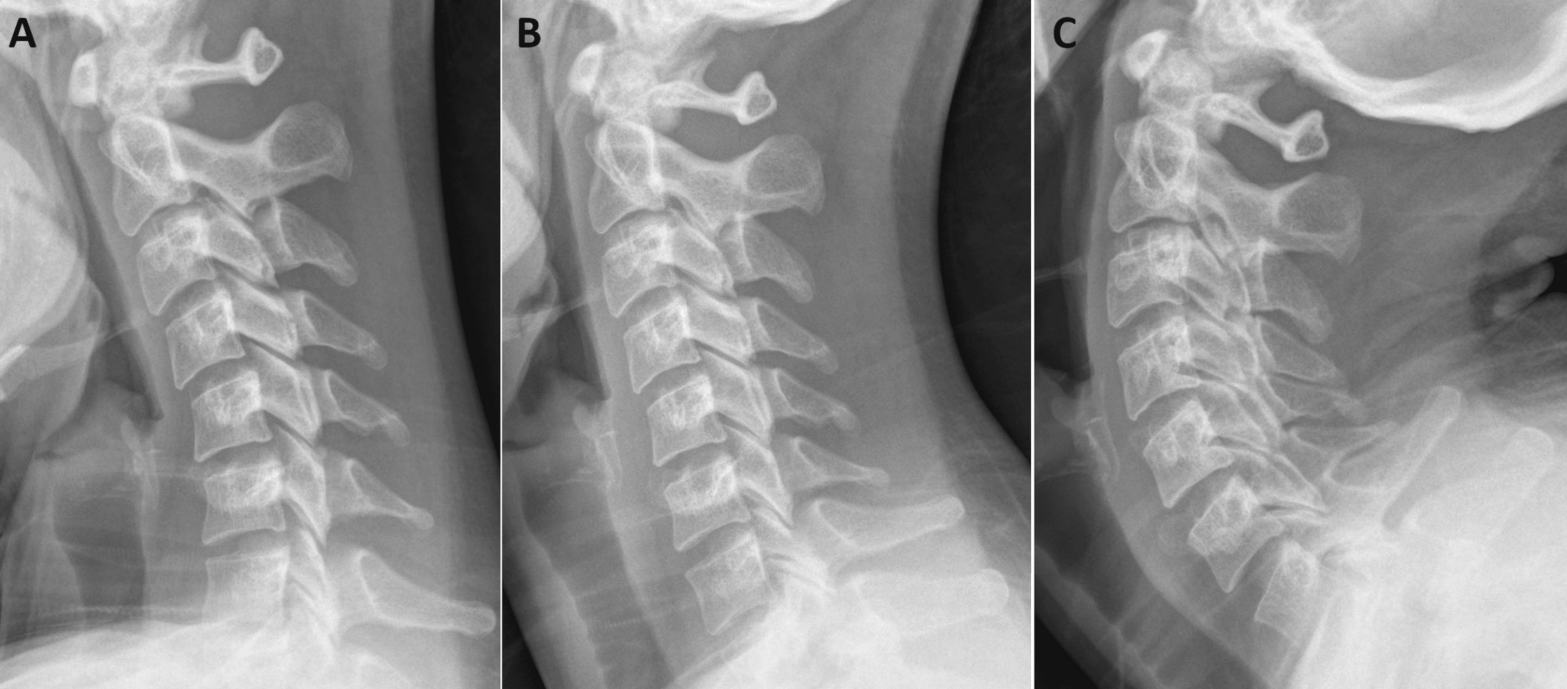
Lateral cervical radiographs taken in the standing position These views illustrate flexion (A), neutral (B), and extension (C). No abnormal listheses or signs of instability or pathology are evident in these views. However, the cervical curve is relatively straight in the neutral position (B).

**Figure 3:**
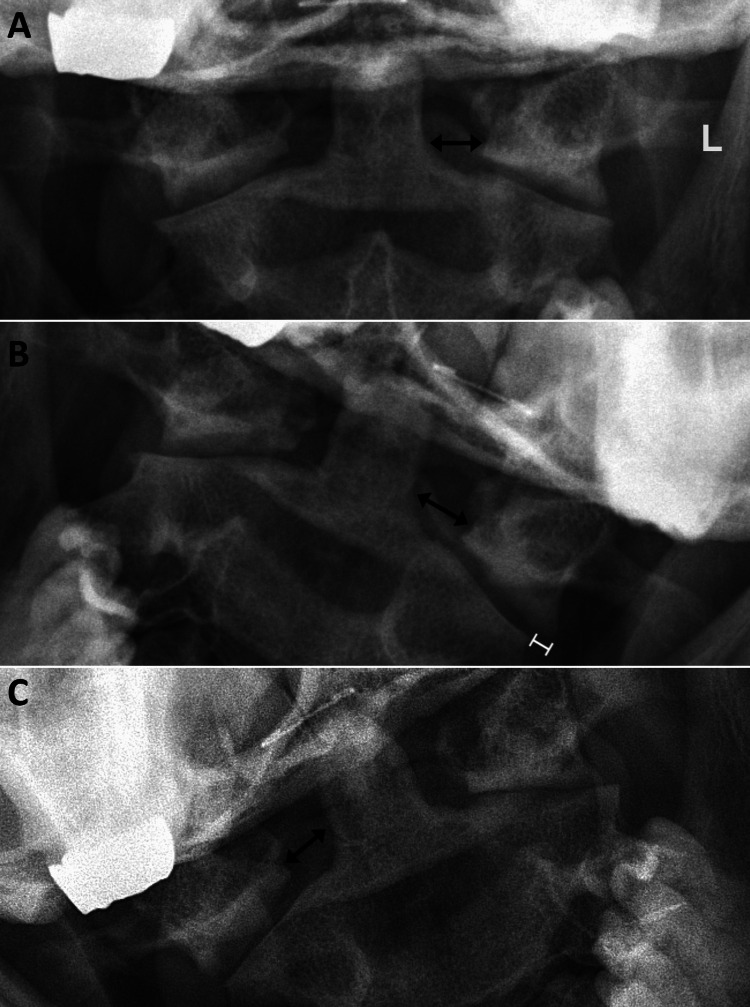
Anteroposterior open mouth lateral bending radiographs The left (L) side marker is shown in the top image, which is consistent across each image (A, B, and C). The neutral image (A) depicts asymmetry of the odontoid-lateral mass interval (OLMI) (double-sided arrows) of 1.7 millimeters (mm) with OLMI measurement larger on the left (7.5 mm) than on the right (5.8 mm). The left lateral bending image (B) demonstrates an increase in OLMI asymmetry to 4.2 mm with OLMI larger on the left (8.0 mm) than on the right (3.8 mm). The right lateral bending image (C) demonstrates an asymmetrical OLMI, albeit smaller in magnitude (7.3 mm on the right, 5.8 mm on the left; asymmetry of 1.5 mm).  A step-off is visible with left lateral bending (B), as the left lateral mass is displaced 2.4 mm to the left relative to the articular pillar of C2.

The chiropractor considered the possibility of cervical instability and post-concussion syndrome and ordered cervical spine radiographs including static and dynamic views. The lateral view demonstrated a straightening of the cervical lordosis (Figure 2), while the anteroposterior open mouth (APOM) view illustrated abnormal lateral translation of C1 on C2 (Figure 3), consistent with a diagnosis of UCI [9,10]. The chiropractor messaged these findings and proposed treatments to the referring cardiologist who concurred with a plan to recommend gentle exercises and manual therapies.

**Figure 4:**
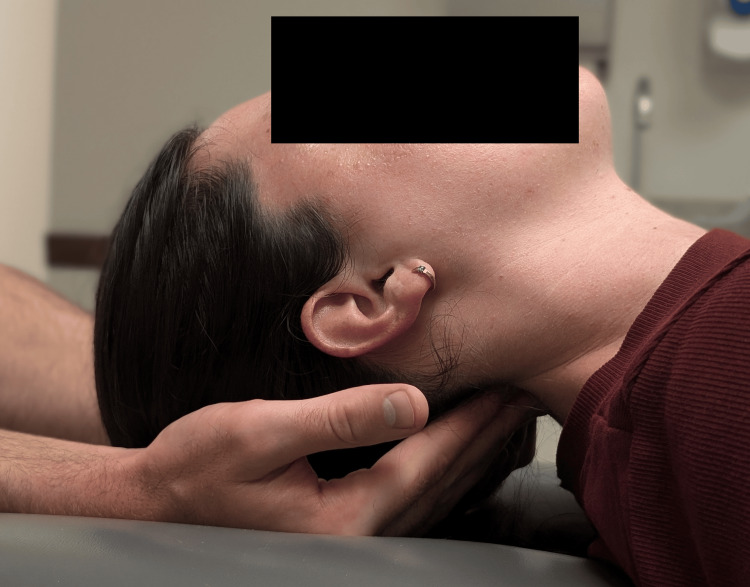
Cervical manual therapy (demonstration) A low-amplitude mobilization using gentle posterior to anterior pressure is applied to the mid-cervical region without the use of a thrust or impulse.

**Figure 5:**
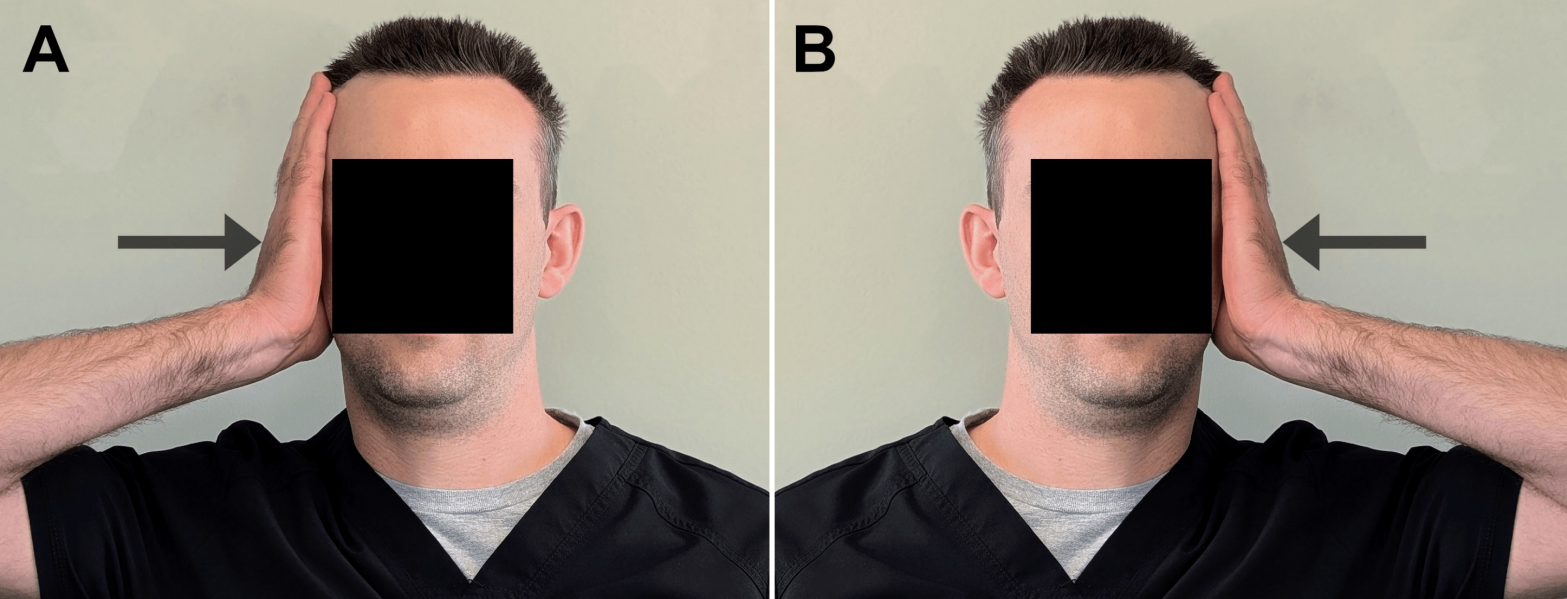
Demonstration of isometric cervical lateral bending exercises The patient places one hand on the right side of the head and applies a gentle, medially directed pressure toward the midline (arrow), while resisting with the neck muscles to maintain a neutral head and neck position (A). This exercise is then performed on the left side (B), with each position being held for 10 seconds, and performed three times per day.

**Figure 6:**
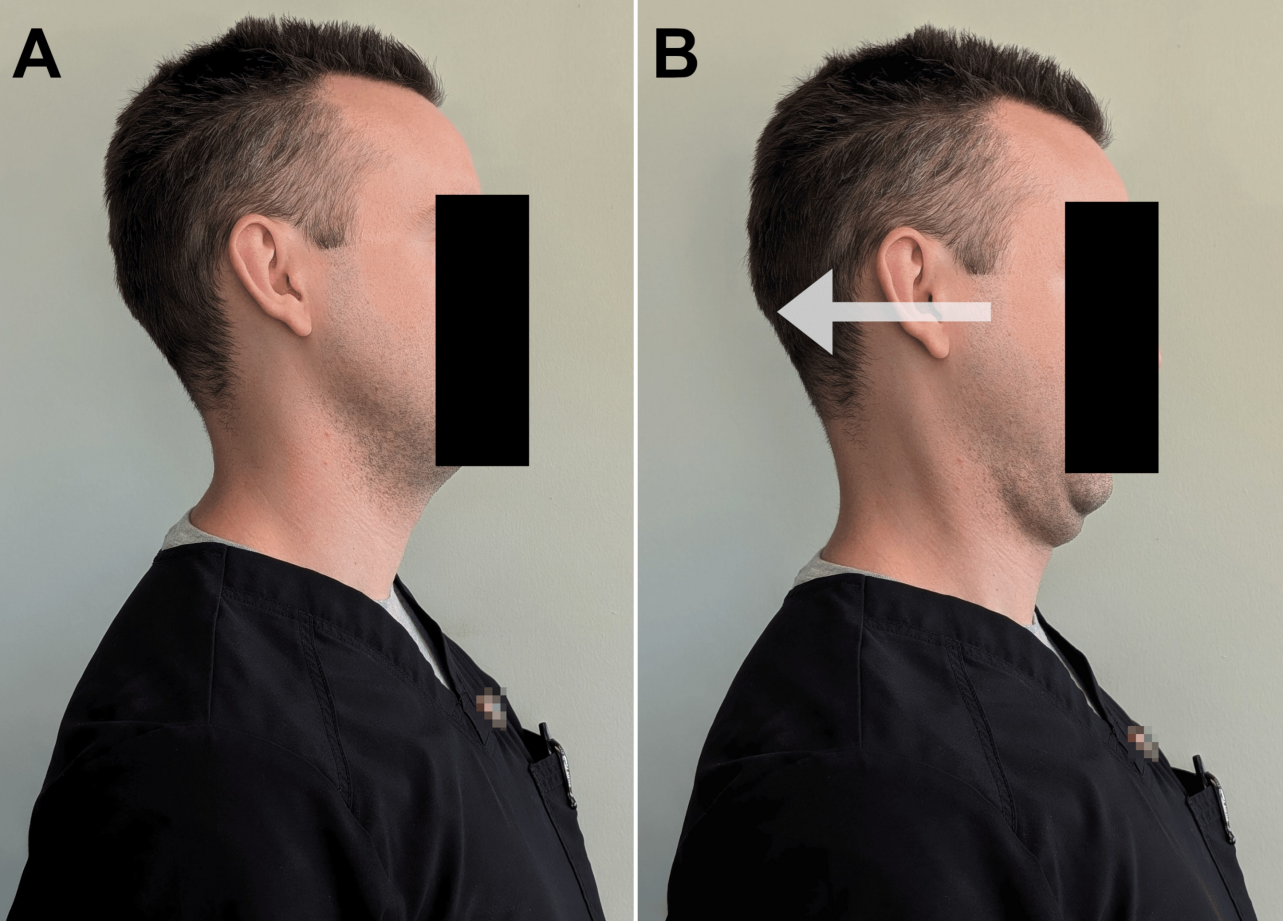
Demonstration of neutral-spine chin retractions (A) The patient maintains a neutral, upright posture with the head and neck. (B) The patient actively moves the head backward (arrow), as if forming a double chin, to engage the deep cervical stabilizers. This exercise is performed for 10 repetitions, three times per day. While this exercise is demonstrated in a standing position, it can also be performed while sitting.

Given the patient’s history of neck pain and dizziness exacerbated by head movements, a targeted approach using manual therapy aimed at stabilizing the cervical spine was deemed appropriate. Upon follow-up with the chiropractor, the patient consented to care including manual therapies used to address the suboccipital and cervicothoracic muscular hypertonicity. These included a supine suboccipital release maneuver, myofascial release, dry needling, and stretches targeting the hypertonic upper trapezius and levator scapulae; posterior-anterior mid-cervical mobilization (Figure 4); and thrust manipulation used in the thoracic spine. All manipulative techniques in the cervical spine were done gently without any thrust/impulse. The patient tolerated all procedures well and noted increasing relief within the days following each visit. The chiropractor demonstrated performing gentle isometric cervical lateral bending exercises (i.e., with opposing hand pressure; Figure 5) and neutral-spine chin retractions (Figure 6) to strengthen the deep cervical stabilizing musculature, to be performed for 10 seconds or repetitions each, respectively, three times per day. The patient was also advised to purchase an Apex® cervical orthosis (Core Products International, Inc., Osceola, Wisconsin) and lie supine with this device under the cervical spine to help restore the cervical lordosis, for two minutes per day. The patient performed home exercises diligently. Treatments were initially provided every two weeks and then progressively spaced apart. 

The patient gradually improved with care, and she began to call into question the necessity of her prescription venlafaxine and requested to her psychiatrist that she be tapered off this medication. The medication was discontinued successfully without recurrence of dizziness symptoms. The psychiatrist noted that there were no signs of panic disorder aside from occasional dizziness, and she no longer required psychiatric intervention. The patient began to start running regularly. By three months, her DHI score had reduced to 24 (moderate handicap). By eight months, her DHI score had reduced to 10 (below threshold for mild handicap). The patient began exercise classes as well. DHI scores are summarized in Table 1.

**Table 1:** Dizziness Handicap Inventory scores Specific dates were avoided for de-identification purposes. Interpretation based on total score: mild handicap, 16-34; moderate handicap, 36-52; severe handicap, 54+.

Measure	Initial	Three months later	Eight months later
Functional subscale	12	10	2
Physical subscale	16	8	6
Emotional subscale	22	6	2
Total	50	24	10

At eight months post-treatment, she only had occasional mild dizziness and no severe episodes of nausea and vomiting nor episodes of being bedridden. By this time, visits had been spaced apart to approximately one month and she had completed 13 chiropractic visits.

## Discussion

The present case highlights a 27-year-old woman with CGD related to underlying lateral UCI and POTS who improved substantially with conservative care including manual therapy, exercises, and increased salt intake. The patient’s presentation is consistent with CGD, given the presence of an underlying cervical spine disorder, considering her dizziness improved following cervical spine treatment, and given that other potential causes of dizziness were excluded [7]. She did not meet the criteria for multiple sclerosis, canalith repositioning maneuvers were ineffective, and post-concussion syndrome was unlikely given the lack of cognitive symptoms. Research suggests that CGD often presents as lightheadedness or imbalance, rather than the rotatory dizziness seen in vestibular disorders [3,11], matching our patient’s presentation. Cervical proprioceptive deficits may have triggered dizziness during her vestibulo-ocular reflex testing; however, it should be noted that this response lacks a definitive diagnostic value in CGD [3].

The patient exhibited both clinical and radiographic features consistent with UCI. Clinically, her history of trauma, preserved cervical range of motion, and a soft end feel on motion palpation suggested possible instability [6]. Radiographically, several findings further supported UCI. Stress radiographs showed a lateral offset of C1 on C2, which worsened with lateral bending [6]. The left odontoid-lateral mass interval (OLMI) in a neutral position of 7.5 mm exceeded the mean value of 3.5 mm (standard deviation = 0.8) in healthy individuals [12]. Additionally, a step-off of more than 2 mm between the C1 and C2 articular pillars provided further evidence of instability [9,10]. Although the diagnostic value of these measurements is not well established, one study of patients with whiplash trauma found that when two or more imaging findings were present such as a lateral overhang exceeding 2 mm and periodontoid asymmetry, there was a specificity of 79% for predicting injury versus absence of injury [10].

According to the radiologic criteria for anterior-posterior UCI in adults, a threshold of over 3 mm for the atlantodental interval in sagittal plane flexion-extension views is concerning for instability [5,6,9]. Conversely, there is no widely accepted consensus for diagnosing lateral UCI [5,6,9]. Therefore, the thresholds for lateral C1 overhang and OLMI mentioned herein should be interpreted on a case-by-case basis, considering the patient’s overall symptoms and clinical presentation. Additionally, diagnosing lateral UCI can be challenging due to its broad and inconsistent symptoms, which range from neck pain and headache to dizziness and various other neurological manifestations [6].

Previous case reports have described the successful use of manual therapies in patients who made limited progress with other treatments for POTS. One recent case report described a resolution of dizziness in a 50-year-old female patient with POTS following a multimodal treatment including cervical and thoracic spinal manipulation [13]. In contrast to the present case, there was no evidence of UCI. Another case report described improvements in POTS symptoms in a 22-year-old female patient with Ehlers-Danlos syndrome after receiving cervical and thoracic mobilization alongside other exercises, such as deep neck flexor strengthening [14]. Again, while there was no evidence of concurrent UCI in that case, the patient had previously undergone C1/2 surgical fusion to treat UCI.

Conventionally, POTS is treated via an individualized approach using non-pharmacologic and/or pharmacologic treatments, rather than manual therapies. Common treatments include increases in salt and fluid intake, compression stockings, and various pharmacologic agents [15]. Considering the present and previous case reports have illustrated the potential utility of an integrative or multimodal approach to POTS, these interventions warrant further research, especially for patients with coexisting cervical spine dysfunction.

In contrast to POTS, manual therapies appear to be more commonly used and supported by evidence for the treatment of CGD [16]. However, the literature is limited when the focus is narrowed to CGD related to lateral UCI. We are aware of one similar case report that described a 32-year-old female patient with dizziness and headache and lateral UCI who responded positively to manual therapies and cervical strengthening exercises [6]. In that case, follow-up imaging was similarly not performed, thereby making it unclear whether manual therapies improved the radiological markers of UCI or solely the symptoms of this condition.

In general, treatments for anterior-posterior and/or rotational UCI (i.e., anterior displacement of C1 on C2) are better described in the literature than those for lateral UCI [17]. Such treatments may include cervical stabilization exercises, bracing, and surgery, which is reserved for cases with myelopathy or individuals who do not respond to conservative interventions [17]. The management and prognosis of this condition are dependent on its severity and relative degree of neural compromise. Furthermore, it remains unclear whether the management of anterior or rotational UCI is identical to that of lateral UCI, which should be a focus of future research. Regardless, authors have encouraged that manual therapies be used cautiously among patients with UCI [17]. As illustrated in the present case, precautions were taken such as avoiding cervical thrust manipulation to ostensibly avoid exacerbating the UCI.

The relationship between POTS and UCI is not well understood, although some authors have speculated that these conditions may be linked, particularly in cases of generalized hypermobility or trauma [18]. One author suggested that UCI may contribute to POTS through vertebrobasilar ischemia, mechanical stress on the brainstem, cerebrospinal fluid (CSF) alterations, or compression of the C2 nerve root(s), which can lead to occipital neuralgia [19]. While our patient did not present with any signs of overt myelopathy or cerebrovascular ischemia, the possibility of less severe functional changes, such as nerve compression or CSF disruption, cannot be ruled out in contributing to POTS. The fact that her POTS symptoms and dizziness did not resolve with prior lifestyle advice and fludrocortisone but substantially improved when her cervical spine was addressed suggests a potential link between these conditions. However, it remains possible that the co-occurrence of POTS and UCI was coincidental.

This case appears unique in that a conservative chiropractic approach was effective in addressing three interrelated conditions: CGD, UCI, and POTS [20]. Traditional chiropractic care for neck pain and headaches often includes spinal manipulative therapy involving high-velocity, low-amplitude thrusts to the cervical joints. However, in cases of UCI, these techniques are avoided due to the potential risks of exacerbating instability [21]. Instead, a gentler regimen was employed, focusing on cervical stability exercises and manual therapies.

Our approach is supported by a case series in which nine patients with UCI responded positively to a regimen centered on cervical extension traction aimed at restoring cervical lordosis [5]. A recent systematic review also found moderate-quality evidence that manual therapy is effective in reducing CGD symptoms, particularly in relation to cervical spine dysfunction [16]. However, given that only a subset of patients with CGD also present with UCI, caution is needed when generalizing findings from studies that primarily include patients with cervical disc disorders or spondylosis [4].

In cases where lateral UCI is suspected, such as in patients with recalcitrant CGD, a neutral APOM view with measurement of the OLMI may be useful in diagnosis. When asymmetry is present or lateral UCI is suspected based on clinical features, APOM lateral bending views may reveal dynamic changes suggestive of lateral UCI [5,6,10]. However, to the best of our knowledge, there are no definitive guidelines on the routine use of dynamic APOM lateral bending views for diagnosing UCI. Positioning can be challenging or limited by a patient’s symptoms or tolerance of the lateral bending position. Therefore, the decision to include APOM lateral bending views should rely on clinical judgment.

This case report’s strengths include a multidisciplinary approach. Extensive diagnostic testing and imaging were performed, and progress was objectively tracked using the validated DHI score. This outcome measure is often used in CGD research and has a minimal clinically important difference of 18 points [3,16], which the present patient exceeded. The long follow-up period of eight months also supports the treatment’s sustained effectiveness. However, there are several limitations. No follow-up imaging was conducted to confirm structural improvement, as the patient’s return to normal activity made it unnecessary. Accordingly, it remains unclear whether the patient’s structural and/or dynamic measurements of lateral UCI improved or rather the symptoms improved independently, or both. The absence of quantitative tests for CGD, such as repositioning error or balance testing, limits objective assessment, although these tests have an unclear validity and/or accuracy [3,7]. The patient’s history of anxiety and venlafaxine use could have confounded symptoms. However, the patient’s improvement before and after discontinuing venlafaxine, lack of panic symptoms at a psychiatrist assessment, and clearance from ongoing psychiatric care suggest the dizziness was not chiefly psychogenic. Finally, GAD-7 and PHQ-9 score assessments were not repeated [22], yet this appeared unnecessary given her improvement. A single case report should not be used to draw conclusions about the effectiveness of multimodal or manual therapies for CGD, UCI, or POTS, and further research is needed on this topic.

## Conclusions

The present case highlights a 27-year-old woman with CGD and associated underlying lateral UCI and POTS whose dizziness improved with conservative care including manual therapy, exercises, and increased salt intake. Although the exact mechanisms and interrelationships among lateral UCI, CGD, and POTS remain unclear, the present case highlights a conservative, multimodal approach that benefited dizziness symptoms ostensibly linked to these conditions. This case highlights the importance of a thorough evaluation and tailored treatment approach to dizziness. Although lateral UCI is a potentially uncommon contributor to CGD, relatively basic tests such as dynamic radiographs may be helpful in identifying this condition after ruling out other etiologies of dizziness. While the conservative care and manual therapies may have facilitated this patient’s recovery, further studies are necessary to examine the efficacy of these treatments for CGD with underlying UCI and/or POTS.
